# Non-*Candida* fungal cholecystitis - an uncommon occurrence even in the right host

**DOI:** 10.1007/s10096-026-05541-1

**Published:** 2026-05-14

**Authors:** Mitchell G. Dumais, Mary M. Czech, Mustafa A. Hyder, Rachel R. Lee, Juan C. Gea-Banacloche, Christopher G. Kanakry, Sun A. Kim, Bindu Adhikari, Matthew D. Surette, Adrian M. Zelazny, Amir Seyedmousavi, Jennifer M. Cuellar-Rodriguez

**Affiliations:** 1 Intramural Clinical Management Operations Branch, Office of the Clinical Director, National Institute of Allergy and Infectious Diseases, National Institutes of Health, Bethesda, MD USA; 2 Transplant Infectious Diseases, Laboratory of Clinical Immunology and Microbiology, Division of Intramural Research, National Institute of Allergy and Infectious Diseases, National Institutes of Health, Bethesda, MD USA; 3 Center for Immuno-Oncology, Center for Cancer Research, National Cancer Institute, National Institutes of Health, Bethesda, MD USA; 4 Laboratory of Pathology, National Cancer Institute, National Institutes of Health, Bethesda, MD USA; 5 Microbiology Service, Department of Laboratory Medicine, Clinical Center, National Institutes of Health, Bethesda, MD USA

**Keywords:** Mold cholecystitis, Disseminated *Lomentospora prolificans*, Invasive fungal infection (IFI), Immunosuppression, Hematopoietic cell transplant (HCT)

## Abstract

Invasive fungal infections (IFI) are a significant cause of morbidity and mortality in hematologic malignancy patients and hematopoietic cell transplant (HCT) recipients. Mold cholecystitis has rarely been described. We present a case of disseminated *Lomentospora prolificans*, that had recrudescence of symptoms with acute cholecystitis. Treatment included a temporary cholecystostomy and administration of the novel antifungal fosmanogepix. At 6 weeks post-HCT, the patient underwent cholecystectomy and completed a course of fosmanogepix, leading to resolution of her infection. Here we review the published literature on non-*Candida* fungal cholecystitis, underlying risk factors and treatment.

## Case presentation

A 28-year-old female with high-risk acute myeloid leukemia (AML) was referred to the National Institutes of Health Clinical Center (NIH-CC) for an experimental HCT due to high-risk AML.

**Fig. 1:**
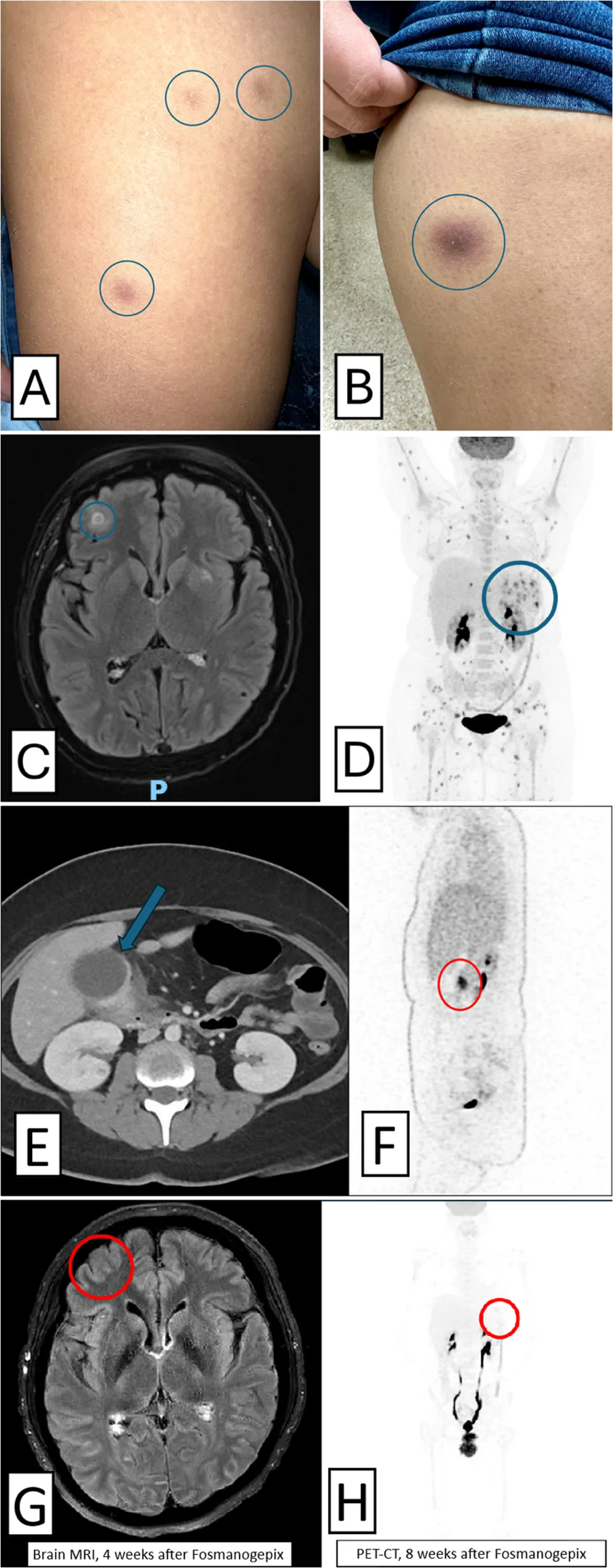
Exam findings on admission to NIH. 1**A**: Subcutaneous nodules noted on presentation to NIH, improved. 1**B**: New enlarging nodule on presentation, right thigh. 1**C**: Brain MRI (FLAIR) demonstrating a right frontal, ring-enhancing lesion (blue circle) and (1**D**) PET-CT noted disseminated lesions, with highlighted splenic nodules (blue circle). 1**E**: CT scan of the abdomen at the time of acute cholecystitis symptoms, demonstrating gallbladder wall thickening and inflammatory changes extending to the duodenum (blue arrow). 1**F**: PET-CT performed as part of surveillance just days prior to fever, which captures developing hyperintense signal at the gallbladder (red circle). 1**G**: Interval imaging with brain MRI 4 weeks after treatment notes resolution of previously seen ring-enhancing lesion (red circle). 1**H**: Repeat PET-CT after 8 weeks shows near complete resolution of disseminated hyperintense areas (red circle)

She was diagnosed with AML > 1 year prior and had achieved four transient remissions. During the hospitalization for the fourth reinduction chemotherapy (prior to transferring her care to our hospital), she developed febrile neutropenia. Blood cultures were obtained with a single aerobic bottle positive for *Wangiella* (*Exophiala*) spp., despite being on posaconazole prophylaxis. Her antifungal treatment was therefore changed to liposomal amphotericin B. Shortly after, she developed multiple painful subcutaneous nodules on the lower extremities. Her central venous catheter was removed; fever resolved and repeat blood cultures showed no growth. After neutrophil recovery, transition to voriconazole was attempted, but discontinued after developing drug-induced liver injury (DILI). When evaluated at our center 4 weeks later, she was afebrile, non-neutropenic, and not on anti-fungal therapy. Physical exam demonstrated multiple painless, faint macules, on her lower extremities (Fig. 1A). Notably one lesion had recently enlarged, and was mildly tender to palpation (Fig. 1B). Due to concerns for progression of a disseminated IFI, further workup was pursued. Brain MRI revealed ring-enhancing lesions with central cavitation (Fig. 1C). A positron emission tomography (PET) demonstrated FDG-avid lesions throughout the musculature, soft tissues, spleen, kidneys and brain (Fig. 1D). Skin biopsy was consistent with panniculitis, though without organisms on fungal stain, and no-growth on culture. Serum beta-D-glucan (BDG) was positive at 116 pg/mL. Cerebrospinal fluid (CSF) showed no abnormalities, including fungal stain, culture, CSF-BDG, and flow cytometry. Bone marrow biopsy showed morphologic remission but still detectable leukemia at 1.4% abnormal myeloblasts. Despite tissue and fluid specimens yielding no organisms, our imaging findings in this neutropenic patient increased our concern for disseminated IFI.

Voriconazole was started for presumed persistent *Exophiala* spp. but transitioned to posaconazole and terbinafine after recurrent DILI. Transplant was delayed for approximately 2 weeks. During this period, marrow blasts progressed to 13%, but she remained non-neutropenic and developed no new skin lesions. Follow-up PET showed improvement in muscular and soft tissue lesions, stable splenic and renal lesions, and slightly increased FDG-avidity in the proximal GI tract abutting the gallbladder of unclear significance at that time (Fig. 1F).

**Fig. 2:**
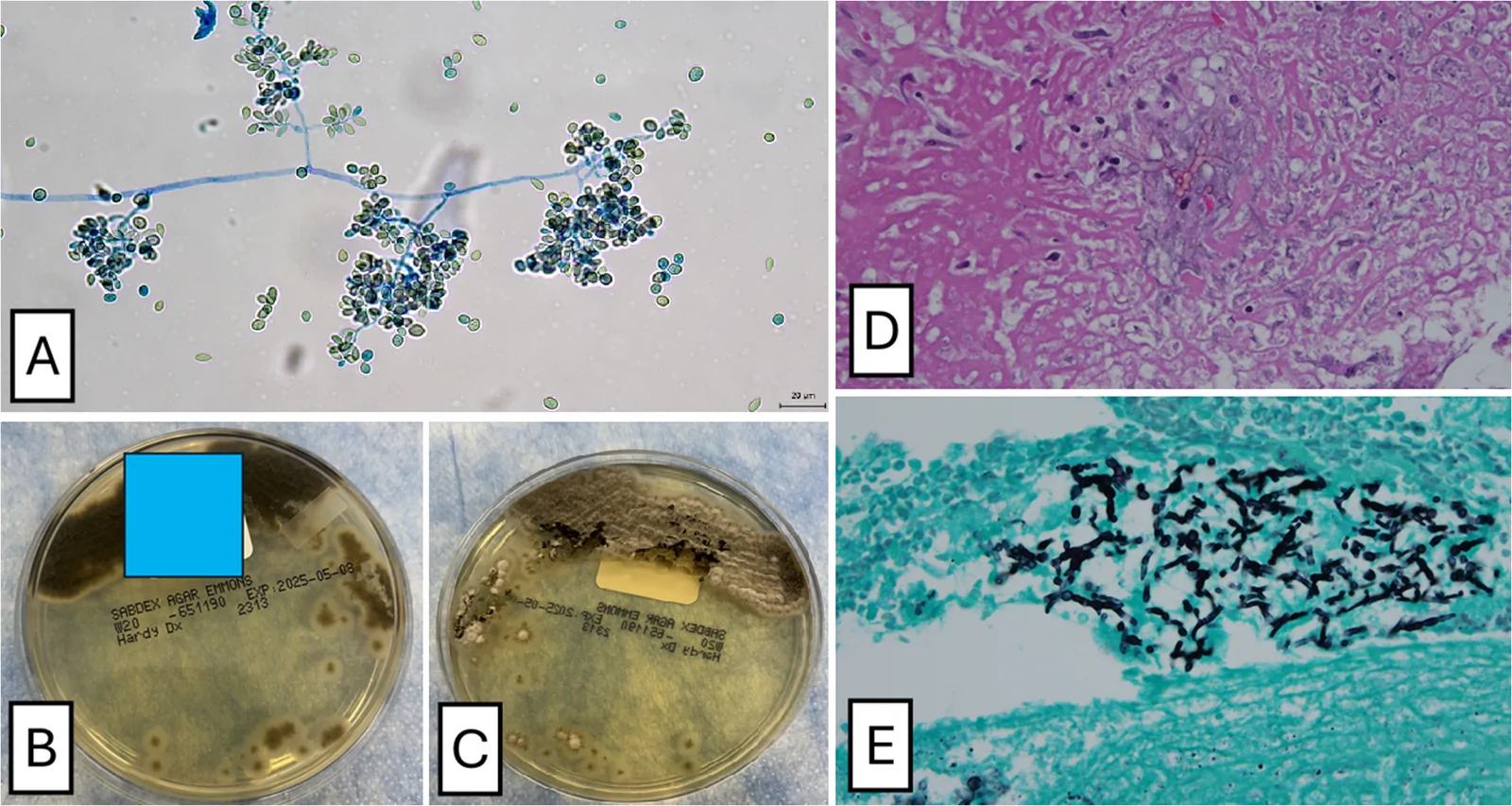
Lomentospora prolificans isolated from our patient at the NIH Clinical Center. 2**A**: Microscopy: Conidiogenous cells arranged in small, brush-like clusters, swollen flask-shaped, dark, slightly inflated conidia (3–7 × 2–5 µm). Scale bar: 20 µm. 2**B**: Colony morphology, reverse view; 2**C**: Colony morphology, front view: Five-day-old colonies grown on Sabouraud dextrose agar at 30°C, exhibiting flat, cobweb-like aerial mycelium with olivaceous grey to blackish coloration. 2**D**: Branching and septate hyphae are identified in necrotic tissue (Hematoxylin-eosin stain, original magnification x400). 2**E**: Grocott's methenamine silver (GMS) stain highlights aggregations of fragmented septate hyphae and conidia

Two days prior to the start of myeloablative conditioning, the patient developed fever prompting a CT, which showed an inflammatory process surrounding the gallbladder and extending to the duodenum (Fig. 1E). Follow-up hepatobiliary iminodiacetic acid (HIDA) scan was consistent with cholecystitis. A cholecystostomy tube was placed. Bile stains revealed many neutrophils, few yeast-like cells, and numerous septate hyphae. Given that the leukemia was progressing, and sustained neutrophil recovery was likely only to be achieved after successful transplant, the decision was made to proceed with conditioning. Growth on bacterial media, fungal media, and nocardia media was identified as *Lomentospora prolificans* (Fig. 2). No other organisms, including bacteria, were isolated from these samples. *L .prolificans* was confirmed by MALDI-TOF (matrix-assisted laser desorption/ionization-time of flight mass spectrometry) and by sequencing of ITS rDNA region [1]. Using the MALDI Biotyper, the NIH Bruker Daltonics database identified the isolate’s protein spectrum as *Lomentospora prolificans*, with spectral scores > 2. The resulting DNA sequences were then aligned against the NCBI GenBank and the International Mycological Association–Westerdijk Fungal Biodiversity Institute databases. Comparison of the concatenated ITS1-ITS2 sequence (~ 500 nucleotides) demonstrated > 99.8% identity with *L. prolificans* type strains. There were no other fungal species within 1% identity. She transitioned to voriconazole, micafungin, and terbinafine, and underwent HLA-haploidentical HCT followed by a prophylactic donor lymphocyte infusion per protocol on D + 7.

*L. prolificans* AFST, performed according to CLSI M38-ED3 guidelines [2], demonstrated reduced in-vitro activity (MEC > 16 µg/mL) for amphotericin B, itraconazole, voriconazole, posaconazole and isavuconazole; terbinafine (> 2 µg/mL), and micafungin (> 8 µg/mL). However, olorofim (0.125 µg/mL) and manogepix (≤ 0.008 µg/mL) had low MEC. Given AFST and the prior reported *Wangiella* spp. infection—an organism with intrinsic resistance to olorofim—an Emergency Investigational New Drug (EIND) application was submitted to the U.S. Food and Drug Administration in collaboration with Basilea Pharmaceutica International Ltd, Allschwil, for fosmanogepix. Following approval on HCT D + 10, IV fosmanogepix 600 mg/day was added after a 24-hour twice-daily loading dose of 1000mg. Due to ongoing DILI, voriconazole and terbinafine were discontinued on D + 14, and continued single agent IV fosmanogepix. She had fevers early post-graft infusion and peri-engraftment, but otherwise remained clinically and radiologically stable, and engrafted on D + 14.

On D + 25 developed nausea and vomiting, and on D + 27 recurrent fevers. On D + 31 had recrudescent positive serum-BDG (peak 175 pg/mL). Repeat bile cultures consistently grew *L. prolificans* with unchanged MEC value for manogepix. On D + 40, developed new RUQ pain and underwent laparoscopic cholecystectomy. Surgical cultures from the gallbladder and a biofilm on the liver grew *L. prolificans*. The gallbladder specimen showed diffuse wall thickening, patches of pale velvety mucosa and an exudate. Microscopic examination revealed fibrotic gallbladder wall, chronic inflammation with eosinophils. Septate hyphae and conidia were identified in the fibrino-inflammatory exudate and necrotic tissue (Fig. 2). Her fever resolved and BDG normalized. The patient recovered clinically and was discharged after transitioning to oral fosmanogepix 800 mg/day. Repeat brain MRI on D + 37 showed resolution of lesions (Fig. 1G). She had complete clinical resolution and negative PET/CT on D + 70 (Fig. 1H). Unfortunately, approximately 100 days after transplant, was admitted with bacterial pneumonia and altered mental status and found to have new brain lesions in the setting of relapsed leukemia. It was unclear whether the brain lesions represented leukemia or infection. Extensive CSF evaluations, including CSF microbial metagenomic sequencing were negative. The brain lesions seemed to respond to steroids, suggesting more likely leukemic in nature. She was discharged to hospice and died shortly thereafter.

Throughout her course, we maintained some skepticism regarding the prior *Wangiella* spp. fungemia. The NIH-CC mycology laboratory was able to obtain the original blood isolate and confirmed—using phenotypic and molecular methods—that it was, in fact, *L. prolificans*, which had been misidentified as *Wangiella* spp. The previous inaccurate organism identification relied primarily on morphological analysis, which has limitations and can be prone to mistakes without definitive molecular diagnosis. The AFST profile on the blood isolate was identical to that obtained from bile cultures.

## Literature review and discussion

*L. prolificans* (formerly *Scedosporium prolificans*) is an emerging opportunistic fungal pathogen primarily found in soil, water, and other environmental sources [3]. After inhalation or inoculation, it can present with a wide array of clinical manifestations ranging from colonization to invasive disease [3, 4]. Disseminated infection primarily occurs in immunocompromised hosts, such as those receiving chemotherapy for malignancy or undergoing HCT, often associated with severe neutropenia [5]. Disseminated infection is multi-visceral and carries high mortality risk in these populations (73% − 91%) [6, 7], commonly involves the lungs, central nervous system, eyes, skin, heart and joints [6].

We believe this to be the first reported case of *L. prolificans* causing cholecystitis. Cholecystitis is uncommonly caused by fungi [8], even in patients with disseminated IFI or fungemic. Most reported cases are caused by *Candida* spp. In a recent review of fungal cholecystitis (1976–2019), 86% of patients had monomicrobial detection of Candida, and remaining cases were polymicrobial, with bacteria associated with the biliary tract [9].

Published cases of non-*Candida* fungal cholecystitis are scant and summarized in Table 1. Most non-*Candida* fungal cholecystitis cases occur in the context of disseminated infection (9/10 cases), likely resulting from hematogenous dissemination. Aside from our case, there are four other cases of proven mold cholecystitis (*Fusarium* (1) and Mucorales (3)). Notably all inherently resistant molds. Like our patient, two of these mold cholecystitis cases occurred as disseminated disease in the setting of AML and neutropenia. One case of Mucorales cholecystitis occurred as disseminated disease following surgical instrumentation, and another case of Mucorales cholecystitis occurred as localized disease concurrent with calculous cholecystitis. Four cases of fungal cholecystitis have been attributed to disseminated endemic mycoses (*Histoplasma* (3), and *Coccidioides* (1)) and one due to cryptococcosis. All patients received antifungals (except treatment not reported for the patient with cryptococcosis), and cholecystectomy or cholecystostomy tube. Patients with disseminated mold infection died, whereas those with localized mold infection survived. Three patients with disseminated endemic mycoses survived. Outcomes are not known for the other patients. Omitted from Table 1 is a report of a lung transplant recipient in whom hyphae were identified on cholecystectomy histopathology; but further details are not provided [10], and a case of *Aspergillus* cholecystitis likely introduced at the time of ERCP [11]. Reports of fungi isolated from the biliary tract and gallstones, without concordant clinical symptoms and/or histopathology evidence of invasive disease were also omitted [12–14].

In our review, we differentiate between fungal cholecystitis and fungal cholangitis, as these are separate clinical entities. Patients with fungal cholangitis [15] more commonly have structural abnormalities from underlying malignancy [16], biliary disease [17], or had received liver transplantation [18]. Reported mortality in this population was 100%.

As highlighted from our review, source control remains a therapeutic cornerstone. For our patient, *L. prolificans* continued to be isolated on serial biliary cultures while the patient was receiving fosmanogepix, with stable MECs and no evidence of emerging resistance. Animal models indicate manogepix is highly concentrated in the bile [19], despite this, antifungal therapy combined with gallbladder decompression was ultimately inadequate in our patient, although served as temporizing measure through the period of aplasia. Lasting source control was only achieved after cholecystectomy. Mold cholecystitis is inherently complicated due to its invasive nature, and early cholecystectomy is preferable when clinically feasible.

Treatment of invasive *L. prolificans* is difficult due to intrinsic resistance to multiple anti-fungals [1, 20], and disseminated disease carries a high mortality risk (73–91%) [6, 7]. Guidelines recommend voriconazole with terbinafine offering potential synergism, appropriate surgical intervention, and optimization of immune status [4, 21]. AFST of a collection of *L. prolificans* isolates at the NIH, showed elevated geometric mean MIC/MEC to all tested antifungals, with the exception of olorofim; notably, manogepix was not among the antifungals tested [1]. Other studies have demonstrated low MICs/MECs for *L. prolificans* against olorofim and manogepix [22–24]. The availability of both intravenous and oral formulations of fosmanogepix makes it an attractive option for patients with gastrointestinal mucositis from chemotherapy or GVHD, as well as for those needing prolonged therapy. Clinical data on the treatment of *L. prolificans* with novel antifungals is still very limited.

**Table 1:** Table of published non-candida fungal cholecystitis cases

Cholecystitis pathogen	Underlying Dx	Disseminated	Treatment	Change in Immune Status	Outcome	Reference	
Molds							
*L. prolificans* (* current report)	AML with Neutropenia, peri-HCT	Yes	Voriconazole, terbinafine, followed by fosmanogepix and cholecystectomy	Neutrophil recovery (Engraftment)	Cholecystectomy with clinical cure; death from relapsed AML		Present Article
*Fusarium* species	AML with Neutropenia	Yes	Liposomal amphotericin B, voriconazole, intravitreal liposomal amphotericin	G-CSF and AML in Remission	Percutaneous cholecystostomy tube with clinical response; death from septic shock due to bacterial infection		[9]
Mucorales	AML with Neutropenia	Yes	Liposomal amphotericin B and cholecystectomy	None reported	Cholecystectomy, death due disseminated mold 6 days following diagnosis		[25]
*Apophysomyces ossiformis* (Mucorales)	Reported immunocompetent, s/p IR guided drainage of RP hematoma infected w/ K. pneumoniae and subsequent manifestations of disseminated Mucorales	Yes	Liposomal amphotericin B and posaconazole, followed by cholecystectomy	None	Cholecystectomy, death due to complications of disseminated mold		[26]
*Syncephalastrum* spp. (Mucorales)	Immunocompetent with acute calculus cholecystitis w/ superimposed invasive mold cholecystitis	No	None reported	None	Cholecystectomy, full recovery		[27]
Endemic Mycoses							
*C. immitis*	Uncontrolled diabetes, DKA on presentation	Yes	Lifelong liposomal amphotericin B	None	Percutaneous cholecystostomy with delayed cholecystectomy with clinical response; lifelong amphotericin infusions		[28]
*H. capsulatum*	AIDS	Yes	Amphotericin B deoxycholate, followed by itraconazole	None	Cholecystectomy, full recovery		[29]
*H. capsulatum*	Renal Transplant	Yes	Liposomal amphotericin B	None	Percutaneous cholecystostomy, full recovery		[30]
*H. capsulatum*	AIDS	Yes	Liposomalamphotericin B, followed by itraconazole	None	Laparoscopic cholecystectomy, No clinical follow-up		[31]
Other Organisms							
*Cryptococcus* spp.	AIDS	Yes	Not reported	Unknown	Cholecystectomy, unknown		[32]

## Conclusion

Non-*Candida* fungal cholecystitis is uncommon, occurs in immunocompromised hosts, and carries high mortality risk, when secondary to disseminated mold infection. Effective management relies on accurate pathogen identification, active antifungals, source control, and immune reconstitution.

## Data Availability

All data underlying this article are available within the article and supporting figures. For additional clinical information, data will be made available upon reasonable request to the corresponding author.

## Declarations

### Clinical trial protocols

002452-C: Emergency Use Fosmanogepix for a Single Patient With Lomentospora prolificans Infection. 000489-C: Phase I/II Study Using Prophylactic Donor Lymphocyte Infusion Early Post-Transplant After Allogeneic Hematopoietic Cell Transplantation Using Post-Transplantation Cyclophosphamide for High-Risk Hematologic Malignancies
